# Improving cognitive impairment classification in Alzheimer’s disease using tabular diffusion augmentation and late fusion deep learning

**DOI:** 10.3389/fbinf.2026.1949507

**Published:** 2026-10-22

**Authors:** Aidan Z. Wang, Xiaoxi Shen

**Affiliations:** 1 Department of Molecular and Cellular Biology, Harvard University, Cambridge, MA, United States; 2 Department of Mathematics, Texas State University, San Marcos, TX, United States

**Keywords:** alzheimer’s disease, APOE, fusion networks, neural networks, single nucleotide polymorphisms, synthetic data, TabDDPM

## Abstract

**Background:**

Alzheimer’s disease is the most common cause of dementia and is often detected after substantial neurodegeneration has occurred. Distinguishing cognitively impaired individuals from cognitively normal ones using genetic and demographic data can help characterize the genetic contributions to impairment. Deep learning can capture complex, nonlinear genetic relationships among these features, but its use in clinical genomics is limited by the small size of patient cohorts.

**Methods:**

We evaluated whether TabDDPM-based synthetic augmentation could improve cross-sectional classification of cognitive impairment using demographic and APOE-region genotype features. From 542 ADNI participants, we created a stratified 433-participant development cohort (80%) and a 109-participant held-out cohort (20%). Eight predictive models were evaluated using repeated stratified 5-fold cross-validation with 10 repeats. The 346 or 347 participants in the outer training partition were divided into three internal TabDDPM subsets: 80% for generator training, 10% for hyperparameter selection, and 10% reserved for internal generator evaluation. Run-specific synthetic samples were added to the outer training partition at 3×, 5×, 7×, and 10× augmentation. The selected predictive model configuration and augmentation ratio were then evaluated on the 109-participant held-out cohort for assessment.

**Results:**

The synthetic data showed similar demographic structure and common-variant genotype distribution to the real cohort. Synthetic augmentation improved mean AUC across nonlinear models. The best-performing model, a categorical late-fusion neural network, achieved a mean cross-validated AUC of 0.781 with 7× augmentation, compared with 0.738 using real data alone. For the 7× versus 0× comparison, the internal cross-validation analysis yielded a raw paired DeLong p-value of 0.0012 and a Holm-adjusted p-value of 0.03. This improvement was observed in the 109-participant held-out cohort: AUC increased from 0.721 to 0.769, with gains in both sensitivity (0.68–0.77) and specificity (0.58–0.66).

**Conclusion:**

These results suggest that synthetic data augmentation can improve deep learning performance in data-constrained clinical genomic classification. The approach does not replace real patient data, but it can help models learn more stable patterns from cohorts with limited sample size. Because the framework uses standard tabular genomic and demographic features, it may extend to other genomic classification tasks where individual-level patient data are limited.

## 1 Introduction

Alzheimer’s disease (AD) is the leading cause of dementia and a major health challenge. The number of people living with dementia worldwide is projected to rise to 139 million by 2050 (World Health Organization, 2021). AD follows a destructive progression, sometimes spanning decades, through the silent accumulation of beta-amyloid plaques and tau neurofibrillary tangles that accumulate without clinical symptoms (Jack et al., 2013). This motivates efforts to better characterize factors associated with cognitive impairment.

Genetic factors are particularly relevant for AD because the disease has a strong inherited component. The apolipoprotein E (*APOE*) ε4 allele is one of the most important genetic risk factors associated with late-onset AD as heterozygous carriers face 3-4 times elevated risk and up to 15 times in ε4/ε4 homozygotes (Liu et al., 2013). The *APOE* locus at 19q13.32 is also biologically complex. The rs429358 variant confers ε4-associated risk, while rs7412 contributes to the protective ε2 allele (Liu et al., 2013). SNP-SNP interactions within the *APOE* gene have been associated with AD-related neuroimaging phenotypes independently of the main *APOE* ε4 effect (Hibar et al., 2015). Non-additive effects recur across AD genetics, having also been reported at loci such as *CLU* and *ABCA7* (Nazarian et al., 2023). These nonlinear intra-locus relationships make the 168-SNP *APOE* region used in this study a biologically motivated setting for examining genetic contributions to cognitive impairment. Capturing such effects may require models capable of representing nonlinear or non-additive relationships between genotype states and cognitive impairment.

With the success of deep learning in computer vision and natural language processing, deep learning has been widely applied in genetic studies as well (Badré et al., 2021; Shen et al., 2022; Alzoubi et al., 2023; Ye et al., 2025; Wang et al., 2025). Compared with classic linear models, deep learning models can capture nonlinear and non-additive relationships between input features and responses, thanks to the universal approximation property (Hornik et al., 1989; Cybenko, 1989; Yarotsky, 2017; 2018; Schmidt-Hieber, 2020). This is particularly relevant for the *APOE* locus, where the risk associated with heterozygous ε3/ε4 status is not simply intermediate between ε3/ε3 and ε4/ε4 genotypes. However, the main barrier to applying deep learning to genomic classification of cognitive impairment is data scarcity. Clinical genomic cohorts are expensive to collect, and restricted by privacy constraints (e.g., the HIPAA privacy rule). The clinical cohorts most relevant to translational AD research often include only a few hundred participants, far below the sample sizes typically required to train neural networks reliably.

In recent years, breakthroughs in generative models, including the large language models (LLMs) (Brown et al., 2020) and diffusion models (Ho et al., 2020), have expanded what is possible with synthetic data (Abdel-Azim et al., 2026). Data augmentation via generative models offers great potential to address the limitation of data scarcity in medical data. In particular, diffusion models learn to generate new samples by corrupting training data with noise and then training a denoising neural network to reconstruct samples from the original data distribution (Ho et al., 2020). Tabular Denoising Diffusion Probabilistic Models (TabDDPM, Kotelnikov et al., 2023) extend this approach to tabular data by applying Gaussian diffusion to continuous features and multinomial diffusion to categorical features to generate mixed-type synthetic records and preserve inter-feature dependencies. This makes TabDDPM well suited for clinical genomic data, which often contain mixed feature types, including categorical SNP genotype states and continuous demographic variables. Diffusion models are also less prone to mode collapse and can represent heterogeneous tabular data more naturally than many earlier generative approaches (Dhariwal and Nichol, 2021). However, the application of TabDDPM to clinical genomic classification in small cohorts remains relatively underexplored.

In this study, we evaluated whether TabDDPM-based synthetic augmentation could improve classification of cognitive impairment from *APOE*-region SNPs and demographic features in a small ADNI cohort. We also tested genotype embeddings that represent each SNP state as a categorical feature rather than a numerical value, allowing the model to learn non-additive genotype-state effects. This design was motivated by the nonlinear allele dosage effects observed in AD, where *APOE* ε4/ε4 homozygotes exhibit disproportionately elevated risk relative to ε3/ε4 heterozygotes rather than a simple additive doubling (Liu et al., 2013; Hostage et al., 2013).

We further formulated the classification target as binary (CN vs. MCI + AD) rather than three-class (CN vs. MCI vs. AD). The reason is that the development cohort contained only 25 AD participants, providing limited data for reliable training and evaluation of a three-class model. The binary formulation addressed the clinically relevant question of whether cognitive impairment was present rather than the severity-staging question, which would require larger numbers of AD participants. The modeling framework is not restricted to binary classification and could be extended to multiclass classification for larger cohorts with adequate representation of each diagnostic class.

Although this study focuses on cross-sectional cognitive impairment classification using *APOE*-region SNPs, the proposed framework is disease-agnostic. Tabular diffusion-based augmentation combined with neural network models may also be useful in other data-constrained areas of clinical genomics, including other neurodegenerative disorders, rare diseases, and cancer genomics.

## 2 Methods

### 2.1 Real data

This study used whole genome sequencing data from 808 individuals who participated in phase 1 and phase 2 studies of the Alzheimer’s Disease Neuroimaging Initiative (ADNI; adni.loni.usc.edu). We extracted SNPs from the *APOE* region on Chromosome 19 (45,409,005–45,412,652). 28 individuals with a sample call rate below 99% were excluded. Variants with a call rate <99% or a Hardy-Weinberg equilibrium p-value < 1e-6 were also removed. After quality control, 780 individuals and 168 SNPs remained. Each SNP was encoded using additive coding: 0 for homozygous major allele, 1 for heterozygous, and 2 for homozygous minor allele. Diagnostic labels were assigned as cognitively normal (CN = 0), mild cognitive impairment (MCI = 1), and Alzheimer’s disease (AD = 2). Diagnostic labels were obtained from the ADNI-derived diagnosis files, which contained one diagnostic label per participant. Diagnostic visit or time-point information was unavailable. We therefore treated each label as the participant’s cross-sectional diagnostic status. The study did not model longitudinal diagnostic change or conversion to Alzheimer’s disease. We removed 238 records that were missing diagnosis labels. Our final analysis cohort consisted of 542 individuals with genotype, diagnostic class, and demographic data, including age, years of education, and gender (Table 1).

**TABLE 1:** Diagnostic class and demographic characteristics of the study cohort (N = 542).

Characteristic	Value
Diagnostic class	
Cognitively normal (CN)	182 (33.6%)
Mild cognitive impairment (MCI)	329 (60.7%)
Alzheimer’s disease (AD)	31 (5.7%)
Gender	
Female	354 (65.3%)
Male	188 (34.7%)
Age, years	
Mean ± standard deviation	73.1 ± 6.7
Median	73.2
Range	55.0–91.4
Education, years	
Mean ± standard deviation	16.2 ± 2.8
Median	16.0
Range	6.0–20.0

The 542 participants were first divided into a 433-participant development cohort (80%) and a 109-participant held-out cohort (20%) by stratified random sampling, preserving the proportions of cognitively normal, mild cognitive impairment, and Alzheimer’s disease participants in each. The development cohort contained 145 CN, 263 MCI, and 25 AD participants; the held-out cohort contained 37 CN, 66 MCI, and 6 AD participants. The 433-participant development cohort was used for TabDDPM development, cross-validation, predictive model training, and model selection. The 109 held-out participants were reserved exclusively for final evaluation and were not used at any stage of model development. Cohort size was determined by the number of ADNI participants with complete *APOE*-region genotype, diagnostic, and demographic data after quality control, rather than by a prospective power calculation. The 433-participant development cohort is small relative to typical deep learning training sets but is representative of the cohort sizes available in clinical genomic research, which is the setting this work targets. The 109-participant held-out cohort was used for primary final evaluation but had limited precision and power for subgroup analyses, such as distinguishing MCI from AD. The 168 SNPs and three demographic variables were used as TabDDPM input features. Diagnostic class was supplied separately as the conditioning label and was not included among the input features.

### 2.2 Synthetic data generation via TabDDPM

We used TabDDPM to generate class-conditional synthetic records. The model input consisted of 171 features: age, years of education, gender, and 168 *APOE*-region SNP genotypes. Age and education were treated as continuous variables, while gender and SNP genotypes were treated as categorical variables. Each SNP was represented as a three-state genotype (0, 1, or 2). Diagnostic status was excluded from the feature set and used as the conditioning label for generation.

To prevent information leakage, TabDDPM was tuned separately within each outer cross-validation run rather than being fitted to the full development cohort. In each run, 80% of participants (346 or 347) formed the outer training partition, and the remaining 20% (87 or 86) formed the outer evaluation partition. The outer training partition was further divided using an 80%/10%/10% split stratified by diagnostic class (CN, MCI, AD) into TabDDPM training, validation, and test subsets. The resulting TabDDPM training subset contained 276 or 277 participants, while the validation and test subsets each contained 35 participants. The validation subset was used for hyperparameter selection. The test subset was reserved for an internal generator-generalization check and was not used for hyperparameter selection or final generator fitting. After hyperparameter selection, the run-specific generator was fitted using only the corresponding 276- or 277-participant TabDDPM training subset. The outer evaluation partition remained completely separate from generator development and was reserved for downstream predictive model evaluation. These participant counts and roles are listed in Table 2.

**TABLE 2:** Participant partitioning used for model development and evaluation.

Partition	Subset	Participants	Role
Full dataset	Development cohort	433	Cross-validation and model development
	Held-out cohort	109	Final predictive evaluation
Outer cross-validation run	Outer training partition	346 or 347	Predictive model and TabDDPM development
	Outer evaluation partition	87 or 86	Cross-validation evaluation across 50 outer runs
Internal TabDDPM split	TabDDPM training subset	276 or 277	TabDDPM fitting
	TabDDPM validation subset	35	Hyperparameter selection
	TabDDPM test subset	35	Reserved for internal generator-generalization check
Final development cohort TabDDPM run	TabDDPM training subset	346	Final TabDDPM fitting
	TabDDPM validation subset	43	Hyperparameter selection
	TabDDPM test subset	44	Reserved for internal generator-generalization check
Final predictive evaluation	Held-out cohort	109	Evaluation after model selection

Within each outer run, the TabDDPM generator hyperparameters were optimized using the Optuna-based procedure (Akiba et al., 2019), with the search restricted to that run’s internal training and validation subsets. The search considered TabDDPM-specific settings, including the learning rate, denoising-network architecture, batch size, and number of diffusion timesteps. For each candidate configuration, synthetic-data utility was evaluated using an MLP classifier and the macro-averaged F1 score across the CN, MCI, and AD classes on the corresponding real validation subset. The score was averaged across five synthetic-sampling seeds and used as the Optuna objective. After the best hyperparameter configuration was selected, the run-specific generator was retrained using only the TabDDPM training subset. The fitted generator then produced a synthetic pool large enough to support the maximum 10× augmentation condition. Fixed random seeds were used for data splitting, model initialization, and synthetic sampling.

For the final development cohort generation run, the 433-participant development cohort was divided into a 346-participant TabDDPM training subset, a 43-participant validation subset, and a 44-participant test subset. Candidate configurations were trained on the training subset and evaluated on the validation subset. Each Optuna trial used a fixed budget of 500 training steps. After hyperparameter selection, the selected configuration was retrained for the full 4,000 steps using only the 346-participant training subset; the validation and test subsets were not used for final TabDDPM fitting. The test subset was reserved for internal check of generative model performance. The tuning settings, run-specific configurations, final generator configuration, and synthetic-pool sizes are summarized in Table 3.

**TABLE 3:** TabDDPM hyperparameter tuning and synthetic data generation settings. The table summarizes the Optuna tuning procedure, candidate hyperparameter values, run-specific generator configurations, and final development cohort configuration.

Parameter	Setting
Hyperparameter tuning procedure	
Optuna trials	50 per outer run
Learning-rate range	0.00001–0.003, log-uniform
Network depth	2, 3, 4, or 6 layers
Hidden-layer widths	128, 256, 512, or 1,024 units
Batch size	64, 128, or 256
Training per trial	500 steps
Diffusion timesteps	100 or 1,000
Gaussian loss	MSE
Run-specific generator configurations	
Configuration files	One selected TOML configuration per outer cross-validation run
Generator training	Inner TabDDPM training subset for that run
Synthetic pool	3,460 or 3,470 records per run
Final development cohort generator configuration	
Network	MLP with hidden dimensions [256, 512, 256]
Dropout	0.30
Scheduler	Cosine
Learning rate	0.0002
Weight decay	0.0001
Batch size	64
Training duration	4,000 steps
Diffusion timesteps	1,000
Synthetic pool size	4,330 for the 433-participant development cohort

TabDDPM modeled the mixed-type data using Gaussian diffusion for the continuous features and multinomial diffusion for the categorical features. For our TabDDPM training, age and education were normalized using quantile normalization and modeled with a Gaussian mean-squared-error loss. Gender and the 168 SNP genotypes were modeled as categorical variables, with SNPs represented as genotype states (0, 1, or 2), preserving their discrete states without additional re-encoding. The generator was conditioned on diagnostic status so that synthetic records could be produced within each diagnostic class. The resulting synthetic records were added only to the corresponding outer training partitions at the prespecified augmentation ratios.

### 2.3 Predictive models

For classification, we formulated a binary outcome of cognitively normal (CN, label = 0) versus cognitively impaired (MCI + AD, label = 1). The mild cognitive impairment (MCI) and Alzheimer’s disease (AD) classes were merged into a single impaired class, directing the analysis toward cross-sectional detection of cognitive impairment rather than disease staging. The 433-participant development cohort consisted of 145 cognitively normal participants (33.5%) and 288 cognitively impaired participants (66.5%; 263 MCI and 25 AD).

The models in the primary comparison used 171 input features: age, education, gender, and 168 SNP genotypes. The predictive models used either numerical or categorical SNP encodings depending on the model configuration. Inverse-frequency class weights were computed from the real participants in each outer training partition and applied during model training.

Predictive model development used repeated stratified 5-fold cross-validation (stratified by diagnostic class) with 10 repeats, resulting in 50 outer training-evaluation runs. In each run, models were trained using the 346 or 347 real participants in the outer training partition, with run-specific synthetic samples added according to the augmentation condition being evaluated. The corresponding outer evaluation partition contained 87 or 86 real participants and was used exclusively for predictive model evaluation; no synthetic samples were added to this partition. Class weights calculated from the real outer-training participants were applied to both real and synthetic training observations.

We evaluated eight model configurations across five model families, from linear baselines to nonlinear tree ensembles and multimodal neural networks. By comparing multiple model families, we examined how performance varied across modeling strategies and genotype representations. The five model families are described below and illustrated in Figure 1.

1. Logistic Regression Baselines. Two logistic-regression models were included as linear benchmarks, both using demographic features and 168 SNP genotypes as numerical values. The primary linear baseline used L2 regularization (Ridge Logistic Regression), which helps control overfitting in the high-dimensional feature set. An additional unregularized logistic regression model, without an L1 or L2 penalty, was included to assess whether the results were sensitive to regularization.
2. XGBoost. Gradient-boosted decision trees were included as a strong nonlinear benchmark for tabular data. XGBoost (Chen and Guestrin, 2016) builds an additive ensemble of shallow decision trees, with each trained to correct the errors from the preceding ones. It processed the genotype and demographic features as numerical inputs and provided a non-deep-learning comparison for the neural models.
3. CatBoost. CatBoost (Prokhorenkova et al., 2018) was included as a second gradient-boosted tree ensemble for its native handling of categorical features. Unlike XGBoost, which treated SNP genotypes as numerical values, CatBoost processed the 168 SNPs as discrete genotype categories (0, 1, 2) without one-hot encoding. Including both tree ensembles allowed a direct comparison of numerical and categorical handling within the same model family.

**FIGURE 1:**
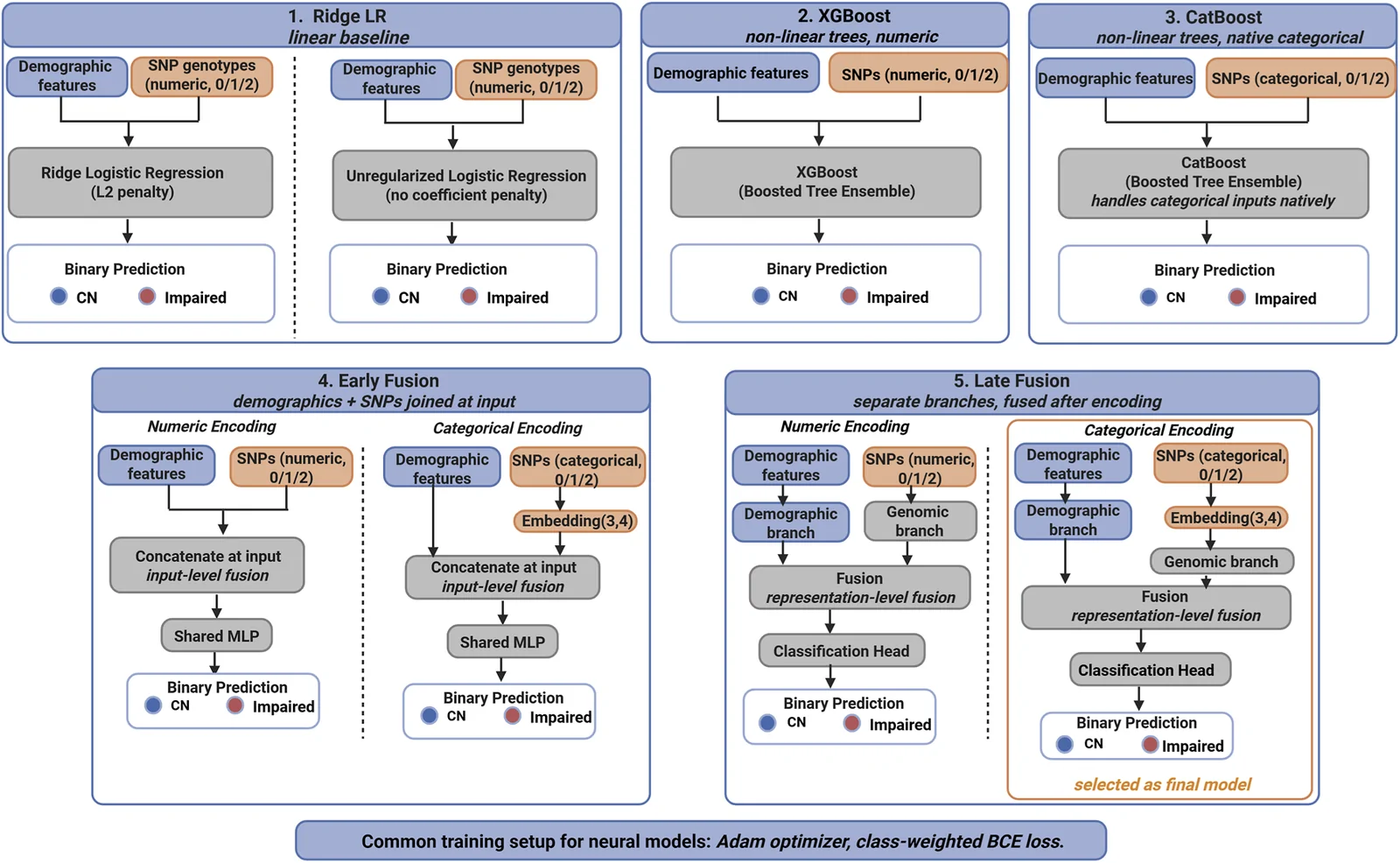
Eight configurations were assessed across five model families. Ridge logistic regression provides the primary regularized linear baseline, and unregularized logistic regression is included as an additional reference model. XGBoost and CatBoost are gradient-boosted tree ensembles, processing genotypes as numerical values and as discrete categories (0, 1, 2), respectively. Early fusion neural networks concatenate demographic and genomic features at the input layer and pass them through a single multilayer perceptron. Late fusion neural networks process demographic and genomic features in separate branches and combine the learned representations at a shared classification head. Early fusion and late fusion are each evaluated with numerical and categorical genotype encodings.

We also considered early fusion and late fusion neural architectures, following standard multimodal learning practice in which information sources are combined either at the input layer or after separate processing (Baltrušaitis et al., 2019; Snoek et al., 2005).

4. Early Fusion. The early fusion model represented the simplest neural integration of the demographic and SNP genomic features. The two feature types were concatenated at the input layer and passed through a single multilayer perceptron with batch normalization and dropout. It produced one joint representation for classification. This architecture tested whether a single shared network could learn a useful, combined representation directly from concatenated inputs.
5. Late Fusion. The late fusion model processed the demographic and SNP genomic features in separate branches before combining them. A demographic branch and a genomic branch each transformed their inputs into intermediate representations of comparable size. These representations were then concatenated and passed to a shared classification head. This design gave each feature type a dedicated sub-network before the two representations were combined. It tested whether learning separate representations for demographic and genotype features improved classification relative to early fusion.

Within both the early fusion and late fusion neural networks, we compared two genotype encodings. In the numerical encoding, each SNP entered the network as its raw genotype value (0, 1, or 2). In the categorical encoding, each genotype state was mapped to a learned embedding. The categorical encoding tested whether learned genotype embeddings could capture non-additive relationships between genotype states better than numerical encoding, which treats genotype values as fixed and equally spaced. The primary configuration used a 4-dimensional embedding. Embedding dimensions of 4, 8, and 16 were evaluated to assess the sensitivity of performance to embedding size.

The categorical late fusion network, the configuration selected for the held-out evaluation, mapped each of the 168 SNP genotypes through a 4-dimensional embedding, producing a 672-dimensional genomic representation. The three demographic features passed through a separate branch of 32 units. The genomic branch reduced its input to 64 and then 32 units, and the two 32-unit representations were concatenated and passed to a shared classification head of 32 units followed by a single sigmoid output. Batch normalization, ReLU activation, and dropout were applied throughout. Figure 2 shows the architecture in detail.

**FIGURE 2:**
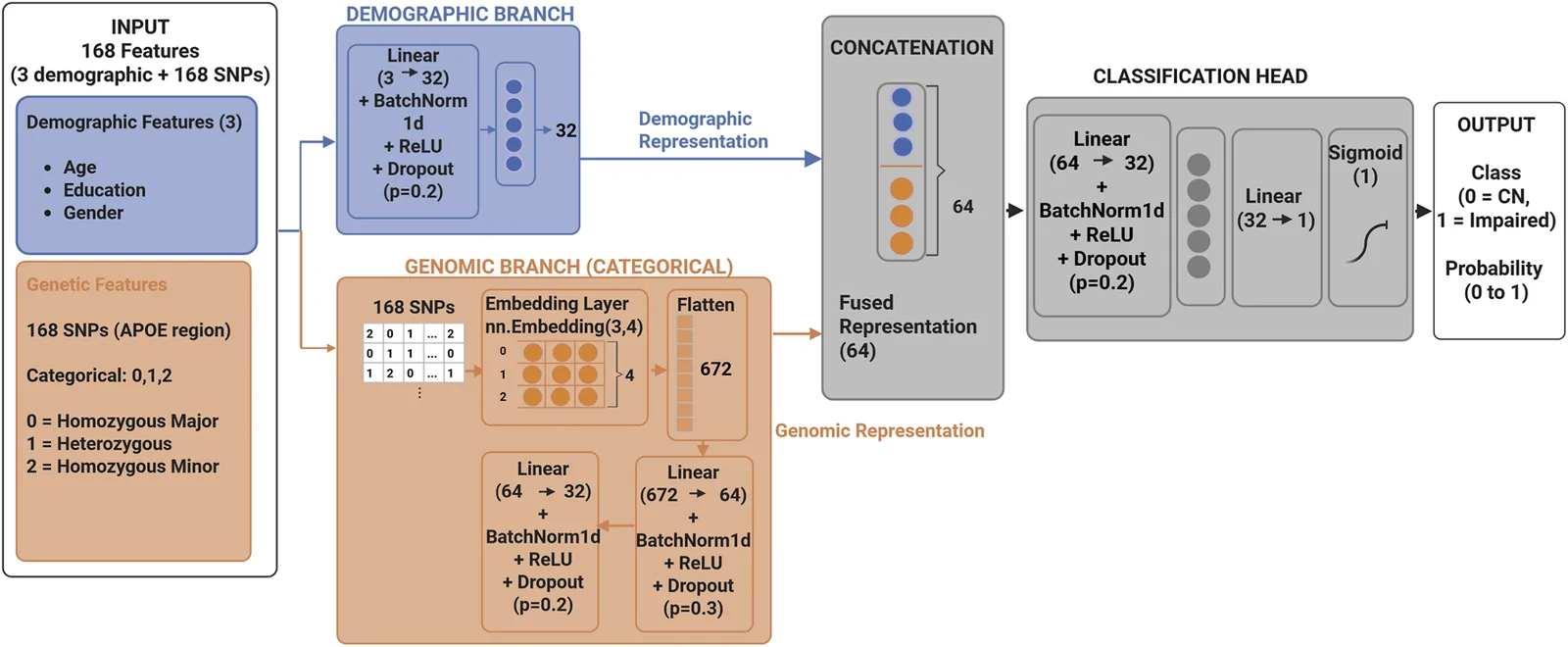
Architecture of the categorical late-fusion model, the best-performing configuration in this study. Demographic and genotype features are processed through separate branches. Each SNP genotype (0, 1, 2) is mapped to a four-dimensional embedding before entering the genomic branch, allowing the model to represent non-additive genotype effects. The two branches produce intermediate representations of comparable size, which are concatenated and passed to a shared classification head for binary classification of cognitively normal versus cognitively impaired.

### 2.4 Computational implementation

The predictive models were implemented in Python. Table 4 summarizes the architecture, genotype encoding, software modules, and training configuration for each of the eight evaluated models. Neural network models were built in PyTorch (Paszke et al., 2019) and trained on CPU within a conda environment. The TabDDPM generative model was implemented using the open-source codebase (github.com/yandex-research/tab-ddpm/). The scikit-learn library (Pedregosa et al., 2011) was used for repeated stratified 5-fold cross-validation, AUC calculation, and logistic-regression baselines, including L2-regularized and unregularized models. XGBoost (Chen and Guestrin, 2016) and CatBoost (Prokhorenkova et al., 2018) were included as non-neural tabular benchmarks, with CatBoost used to evaluate native categorical handling of SNP genotype states.

**TABLE 4:** Model architecture and key implementation details for each evaluated model. For each configuration, the table lists the architecture type, how SNP genotypes are encoded (numerical values or discrete categories), the software modules used, and the training settings applied. For categorical neural models, “Embedding (3, 4)” indicates that the three genotype states (0, 1, and 2) were mapped to a four-dimensional embedding. The table reports the primary configuration; embedding dimensions of 4, 8, and 16 were evaluated separately in a sensitivity analysis.

Model	Architecture	Encoding	Key modules	Training setting
Ridge logistic regression (baseline)	L2-regularized linear classifier	Numerical (0/1/2)	Sklearn LogisticRegression	L2 regularization; C = 1.0; liblinear solver; max_iter = 2000
Unregularized logistic regression (reference model)	Linear classifier without coefficient penalty	Numerical (0/1/2)	Sklearn LogisticRegression	penalty = None lbfgs solver; max_iter = 2000
XGBoost	Gradient-boosted trees	Numerical (0/1/2)	xgboost: XGBClassifier	300 estimators; max depth = 4; learning rate = 0.05; subsample = 0.8; colsample_bytree = 0.8; histogram tree method
CatBoost	Gradient-boosted trees	Categorical (0/1/2)	catboost: CatBoostClassifier	300 iterations; depth = 4; learning rate = 0.05; native categorical SNP handling
Early fusion (numeric)	Single MLP; demographics + SNPs concatenated at input Joint input 171 → 128 → 64 → 1	Numerical (0/1/2)	PyTorch Linear (128) → BatchNorm (128) → ReLU → dropout (p = 0.30) → Linear (64) → BatchNorm (64) → ReLU → dropout (p = 0.20) → Linear (1) → sigmoid	Adam; learning rate = 5 × 10^−4^; weight decay = 1 × 10^−4^; batch size = 64; 80 epochs; weighted binary cross-entropy
Early fusion (categorical)	Single MLP; demographics + 4-Dimensional SNPs concatenated at input Joint input 675 → 128 → 64 → 1	Categorical (0/1/2), embedding (3, 4)	PyTorch Embedding (3, 4) → Linear (128) → BatchNorm (128) → ReLU → dropout (p = 0.30) → Linear (64) → BatchNorm (64) → ReLU → dropout (p = 0.20) → Linear (1) → sigmoid	Adam; learning rate = 5 × 10^−4^; weight decay = 1 × 10^−4^; batch size = 64; 80 epochs; weighted binary cross-entropy
Late fusion (numeric)	Two-branch: Demographics and SNPs processed separately, then joined at a shared head demographic branch 3 → 32; genomic branch 168 → 64 → 32; concatenation 32 + 32 = 64; classification head 64 → 32 → 1	Numerical (0/1/2)	PyTorch demographic branch: Linear (3 → 32) → BatchNorm (32) → ReLU → dropout (p = 0.20) Genomic branch: Linear (168 → 64) → BatchNorm (64) → ReLU → dropout (p = 0.30) → Linear (64 → 32) → BatchNorm (32) → ReLU → dropout (p = 0.20); concatenate; classification head: Linear (64 → 32) → BatchNorm (32) → ReLU → dropout (p = 0.20) → Linear (1) → sigmoid	Adam; learning rate = 5 × 10^−4^; weight decay = 1 × 10^−4^; batch size = 64; 80 epochs; weighted binary cross-entropy
Late fusion (categorical)	Two-branch: Demographics and SNPs processed separately, then joined at a shared head demographic branch 3 → 32; genomic branch Embedding (3, 4) → 672→ 64 → 32; concatenation 32 + 32 = 64; classification head 64 → 32 → 1	Categorical (0/1/2), Embedding (3, 4)	PyTorch demographic branch: Linear (3 → 32) → BatchNorm (32) → ReLU → dropout (p = 0.20) Genomic branch: Embedding (3, 4) → Linear (672 → 64) → BatchNorm (64) → ReLU → dropout (p = 0.30) → Linear (64 → 32) → BatchNorm (32) → ReLU → dropout (p = 0.20); concatenate; classification head: Linear (64 → 32) → BatchNorm (32) → ReLU → dropout (p = 0.20) → Linear (1) → sigmoid	Adam; learning rate = 5 × 10^−4^; weight decay = 1 × 10^−4^; batch size = 64; 80 epochs; weighted binary cross-entropy

Neural network models were trained using the Adam optimizer with a learning rate of 5 × 10^−4^, weight decay of 1 × 10^−4^, and batch size of 64. Binary cross-entropy loss was applied with inverse-frequency class weights to account for class imbalance. Reproducibility was supported by fixed random seeds applied to Python’s random module, NumPy, PyTorch, data splitting, model initialization, and synthetic sampling.

### 2.5 Training and evaluation

#### 2.5.1 Cross-validation and model selection

The overall training and evaluation workflow is summarized in Figure 3. All model configurations were compared on the 433-participant development cohort using stratified 5-fold cross-validation (CV) with 10 repeats (50 runs total). In 5-fold cross-validation, each run splits the 433 participants into an outer training partition (80%, 346 or 347 participants) and an outer evaluation partition (20%, 87 or 86 participants). The same outer training–evaluation splits were used for every model and augmentation condition.

**FIGURE 3:**
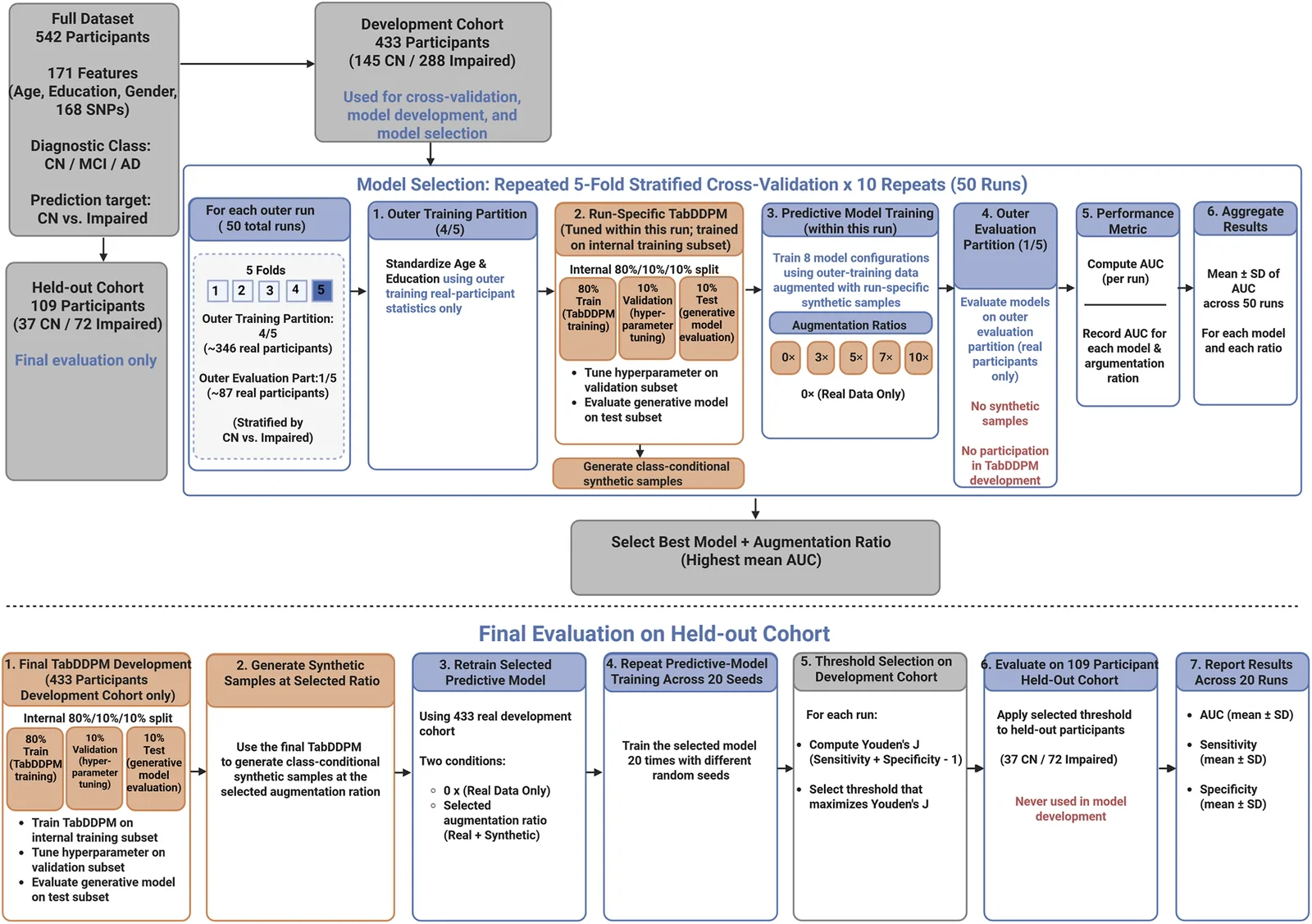
Training and evaluation workflow. The 542-participant cohort was split 80/20 into a development cohort (433) and a held-out cohort (109; 37 cognitively normal; 72 cognitively impaired) reserved for final evaluation only. The classification target was binary (cognitively normal vs. cognitively impaired). Above the dashed line, model selection used 5-fold stratified cross-validation repeated 10 times (50 runs) on the development cohort, adding synthetic samples to the training partition at ratios of 0×, 3×, 5×, 7×, 10× and selecting the architecture and ratio with the highest mean AUC. Below the dashed line, the selected model was re-trained on 433 participants (real-only and augmented) across 20 random seeds, with the classification threshold set on the development cohort by maximizing Youden’s J and applied to the held-out cohort. AUC, sensitivity, and specificity are reported as mean (SD).

All preprocessing and synthetic data generation were performed independently within each outer cross-validation run. For predictive model training, age and education were standardized within each run using means and standard deviations from the real participants in the outer-training partition only. TabDDPM was also trained independently using only participants in that training partition, as described in Section 2.2. The resulting run-specific generator was then used to produce synthetic samples for augmentation of that run’s predictive model training data. Participants in the outer evaluation partition were not used to calculate the mean or standard deviation for feature standardization and contributed neither to TabDDPM training, hyperparameter selection, nor synthetic-data generation, thereby preventing information from the outer evaluation partition from entering the model-development process.

Each predictive model configuration was then evaluated across augmentation ratios of 0×, 3×, 5×, 7×, and 10×, where the ratio denotes the number of synthetic samples added per real sample, and 0× corresponds to real data only. For each outer cross-validation run, synthetic samples generated from the TabDDPM pool were added only to the training partition, so that the training partition was a mixture of real and synthetic participants while the evaluation partition used real participants only.

Model performance was summarized as the mean and standard deviation of the area under the receiver operating characteristic curve (AUC) across the 50 outer evaluation runs. For each model and augmentation condition, the 10 out-of-fold predictions obtained for each development participant were averaged across repeats. Paired DeLong tests compared each augmentation condition with the corresponding real-only (0×) condition. The resulting p-values from the 32 prespecified augmented versus real-only comparisons were adjusted for multiple testing using Holm’s procedure.

Additional comparison analyses were conducted using the same 50 outer cross-validation runs. These included a demographics-only L2-regularized logistic regression model using age, education, and gender; an SNP-only L2-regularized logistic regression model using 168 SNPs without demographic features; and RandomOverSampler from the imbalanced-learn package as a non-generative class-rebalancing control (Lemaître et al., 2017). Within each outer training partition, RandomOverSampler sampled minority-class participants with replacement until the two classes were balanced, without generating new feature values. The selected predictive model architecture was then trained on the resampled data and evaluated on the corresponding untouched outer evaluation partition.

#### 2.5.2 Held-out evaluation

Following cross-validation, the augmented predictive model configuration and augmentation ratio with the highest mean cross-validated AUC were selected for final evaluation. The selected model was retrained on the 433-participant development cohort under two conditions: real data alone (0×) and real data plus synthetic samples at the selected augmentation ratio. Synthetic samples for this final evaluation came from the separate final TabDDPM developed from the 433-participant development cohort, which was internally divided into 346 training, 43 validation, and 44 test participants. Hyperparameters were selected using the validation subset, after which the selected TabDDPM configuration was fitted on the designated 346-participant training subset and used to generate the synthetic samples for final predictive model augmentation. In both final conditions, age and education were standardized using the mean and standard deviation from the 433 real development participants, and inverse-frequency class weights were computed from the real development cohort. Synthetic samples in the augmented condition were transformed and weighted using those same real-development parameters.

The 109-participant held-out cohort consisted of 37 CN participants and 72 impaired participants (66 MCI and 6 AD). The held-out cohort was not used for TabDDPM development or synthetic data generation, hyperparameter selection, predictive model selection or training, or threshold selection, and was used only for final evaluation.

The selected predictive model was retrained 20 times using different random seeds. Classification thresholds were determined separately for the real-only and augmented conditions using only development cohort out-of-fold predictions generated during repeated 5-fold cross-validation within the 433-participant development cohort. Each development cohort participant was evaluated once per cross-validation repeat, yielding 10 out-of-fold predicted probabilities per participant, which were averaged to obtain a single out-of-fold probability. For each condition, the threshold was selected by maximizing Youden’s J statistic (sensitivity + specificity −1), then fixed before held-out evaluation and not re-optimized using held-out predictions. AUC, sensitivity, and specificity were reported as the mean and standard deviation across the 20 retraining runs, with the standard deviation representing variability across model retraining runs.

Participant-level resampling uncertainty was quantified using 5,000 paired stratified bootstrap resamples of the 109 held-out participants. For each bootstrap replicate, the same resampled participants were used for the real-only and augmented conditions, and the observed class counts were preserved. The development-derived classification thresholds remained fixed throughout the bootstrap analysis. AUC, sensitivity, and specificity were calculated separately for each of the 20 seed-specific prediction sets and then averaged across seeds. Bias-corrected and accelerated (BCa) 95% confidence intervals were calculated for each condition and for the paired augmented-minus-real differences using leave-one-participant-out jackknife values for the acceleration term (Efron, 1987).

## 3 Results

### 3.1 Synthetic data quality evaluation

#### 3.1.1 Fidelity of demographic and diagnostic features

For descriptive fidelity assessment, the final synthetic pool was compared with the full 433-participant development cohort with respect to demographic distributions, gender composition within diagnostic classes, and SNP and genotype frequencies. The development cohort consisted of 145 CN (33.5%), 263 MCI (60.7%), and 25 AD (5.8%) participants. TabDDPM generated samples conditionally by diagnostic class, and the synthetic class proportions were specified to follow the corresponding development cohort distribution (Figure 4). Therefore, the similarity in diagnostic class proportions reflects the class-conditional sampling design rather than an independent measure of generative fidelity.

**FIGURE 4:**
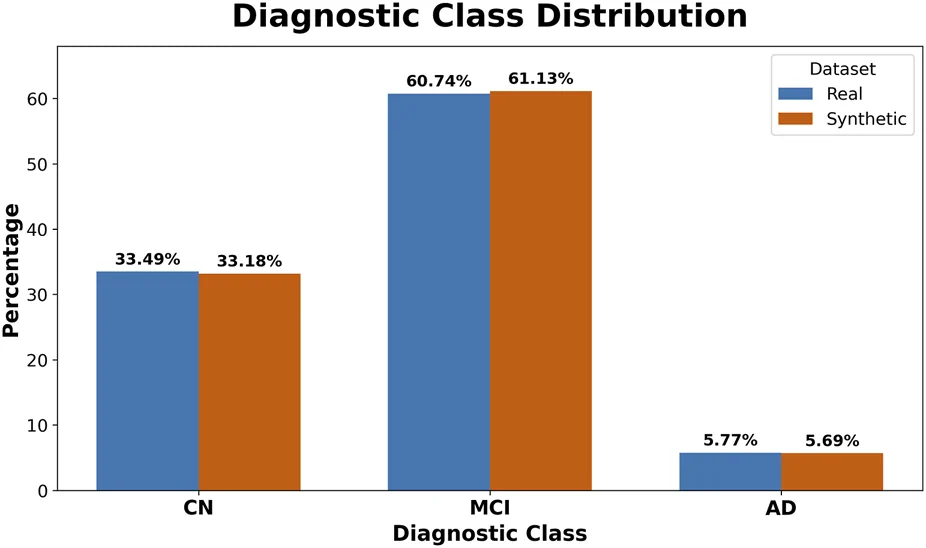
Diagnostic class distribution in real (433-participant development cohort) and synthetic cohorts. Bars show the percentage of participants in each diagnostic class: cognitively normal (CN), mild cognitive impairment (MCI), and Alzheimer’s disease (AD). Real data (blue) and synthetic data (orange). Synthetic samples are generated class-conditionally to follow the development cohort distribution.

Age and education were well reproduced. The kernel density curves for the real and synthetic data overlapped closely across the full range of both variables (Figure 5). The quantile-quantile plots also followed the identity line from the lower to the upper tail (Figure 6). These results show that the synthetic data captured not only the average values of age and education, but also their spread and overall distributional shape.

**FIGURE 5:**
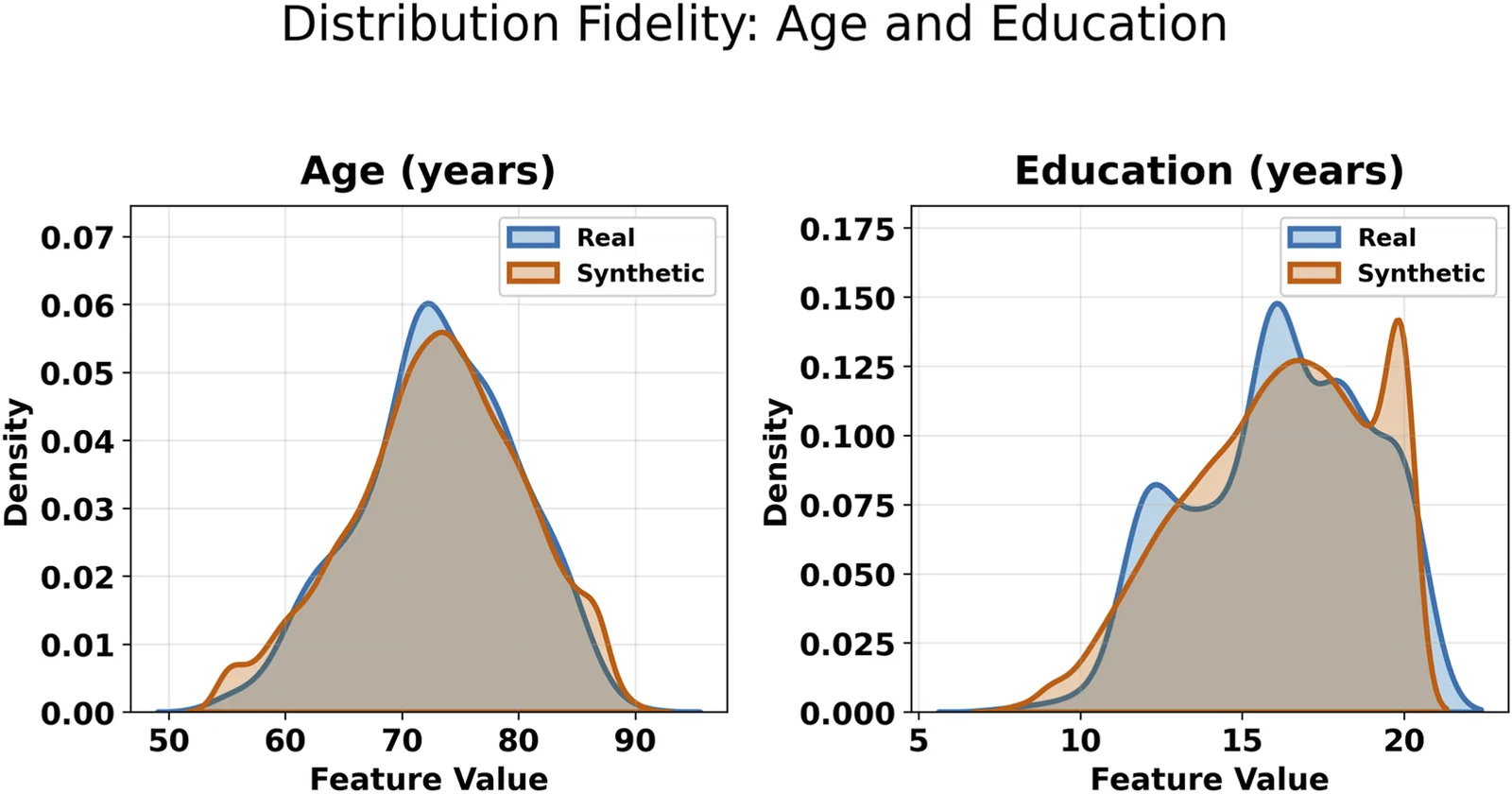
Distribution fidelity for the continuous demographic variables of age and education. Kernel density estimates are shown for real data (blue) and synthetic data (orange). The close overlap between the real and synthetic density indicates the model accurately reproduced the univariate distributions of both variables.

**FIGURE 6:**
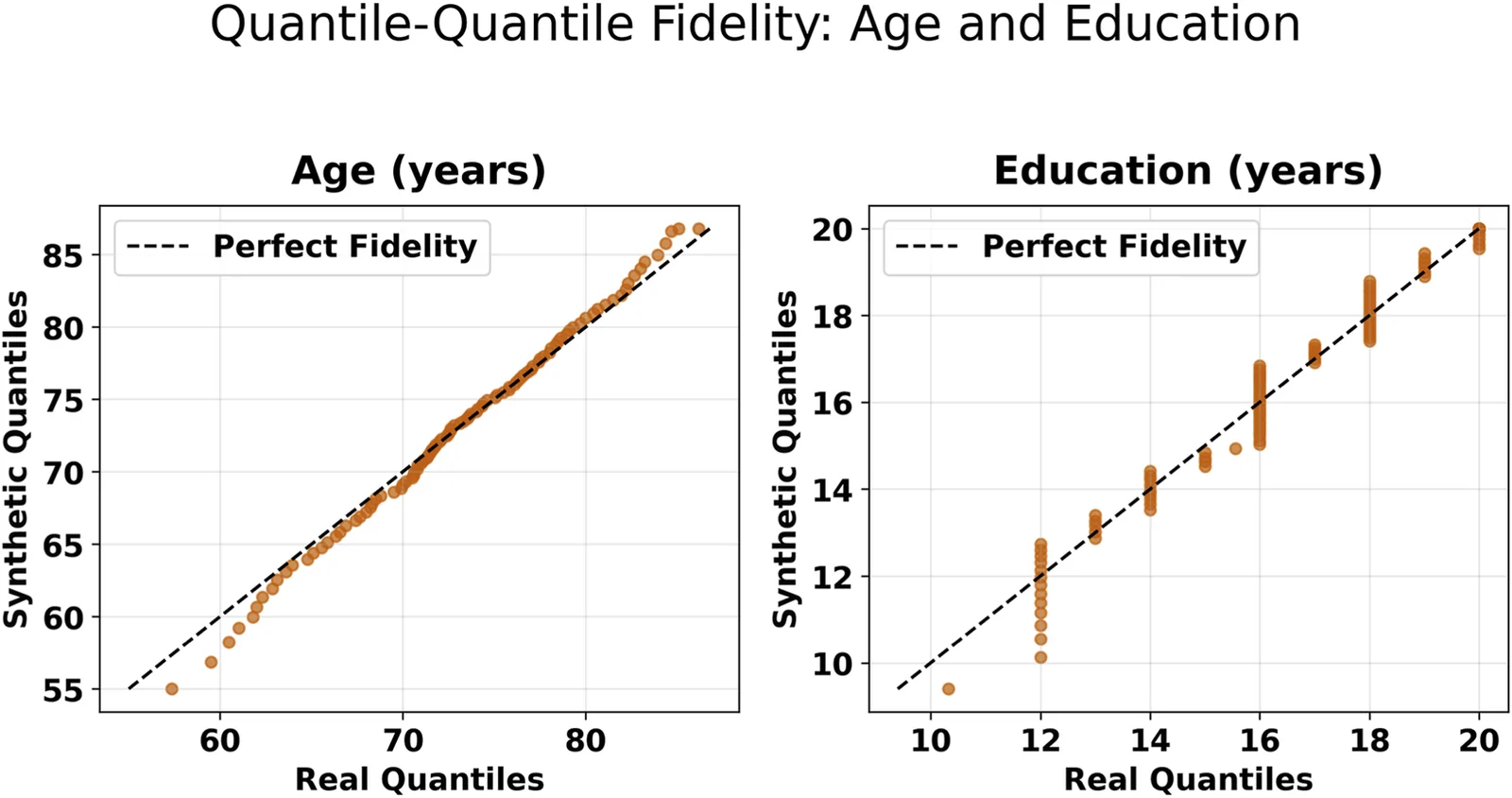
Quantile–quantile comparison of synthetic versus real distributions for age and education. The dashed line indicates perfect alignment between the two distributions. Points close to this line show that the synthetic data reproduced the real distributions across the full range of values, from the lower to the upper tail. The vertical banding in the education panel reflects the discrete integer-year coding of education.

We then evaluated whether synthetic data preserved relationships between gender and diagnostic class by comparing gender composition within each diagnostic group. The male-to-female ratio in the synthetic data closely matched the real development cohort across CN, MCI, and AD participants (Figure 7). This indicates that TabDDPM preserved the observed gender–diagnosis composition in the synthetic data.

**FIGURE 7:**
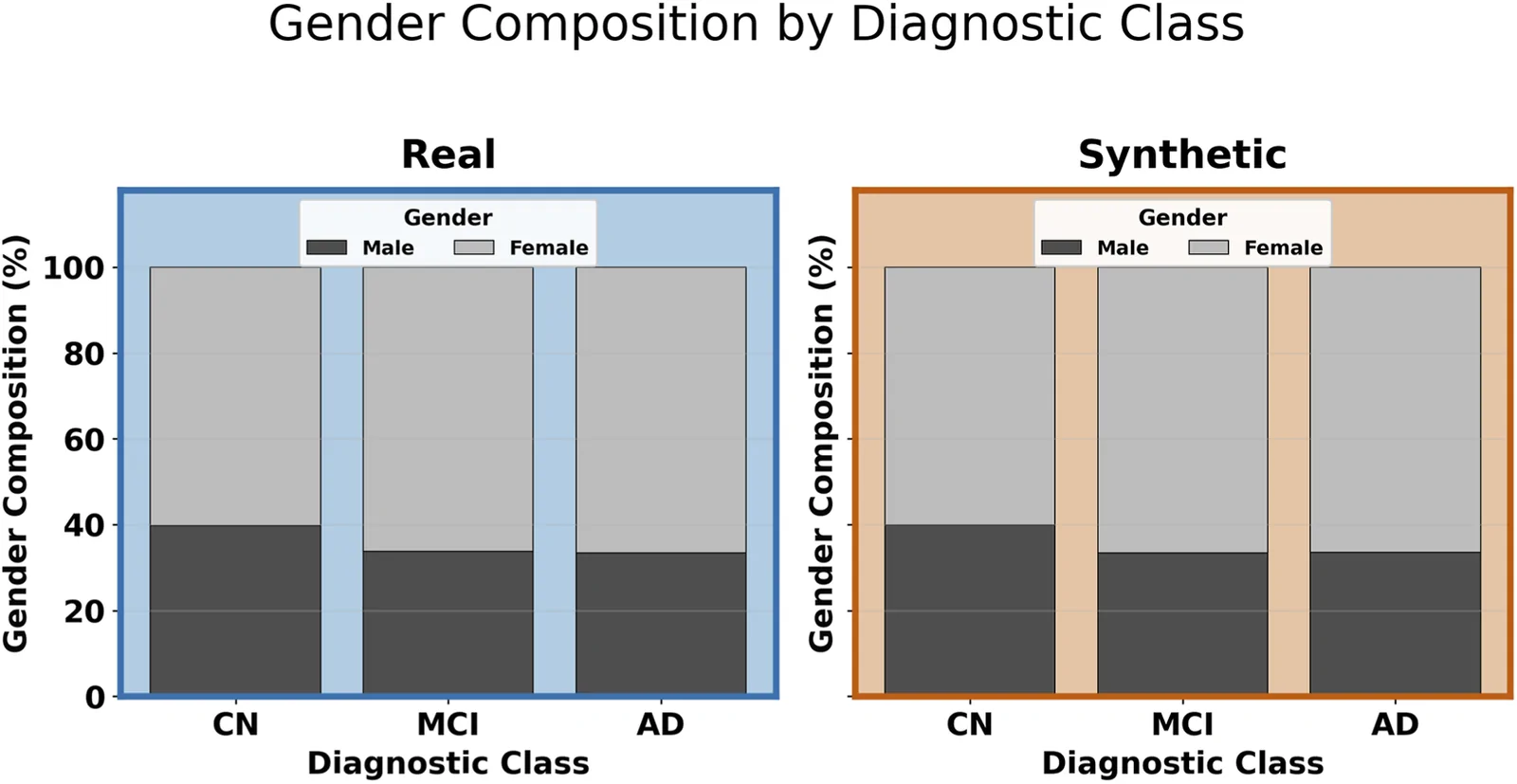
Gender composition within each diagnostic class in real and synthetic data. Real data are shown on the left and synthetic data on the right. Dark grey denotes male participants, and light grey denotes female participants. The within-class gender ratios were similar to those in the real data, consistent with preservation of the observed gender–diagnosis composition.

Overall, TabDDPM reproduced the demographic structure of the real 433-participant development cohort, including the distributions of age and education and the relationship between gender and diagnosis.

#### 3.1.2 Fidelity of SNP features

TabDDPM reproduced common SNP variants more reliably than rare variants. For variants with a minor allele frequency (MAF) of at least 5% in the real cohort, the model kept 26 of 31 SNPs (84%) polymorphic (Table 5; Figure 8). For these retained variants, the proportions of the three genotype states were consistent with their corresponding allele frequencies, indicating that the model preserved plausible genotype patterns. This is important because common variants provide the most consistently represented genetic information.

**TABLE 5:** Synthetic data fidelity by minor allele frequency (MAF) band. MAF bands are defined using allele frequencies in the 433-participant development cohort. Capture rate refers to the proportion of SNPs in each band that remained polymorphic in the synthetic data. All 168 SNPs were retained as model inputs; 69 were monomorphic in the development cohort and are therefore not included in the MAF-band analysis shown here.

MAF band	SNPs (real)	% Of polymorphic panel	Captured	Capture rate
Common (MAF ≥5%)	31	31%	26/31	84%
Low-frequency (1% ≤ MAF <5%)	16	16%	9/16	56%
Rare (0.5% ≤ MAF <1%)	2	2%	0/2	0%
Ultra-rare (MAF <0.5%)	50	51%	0/50	0%

**FIGURE 8:**
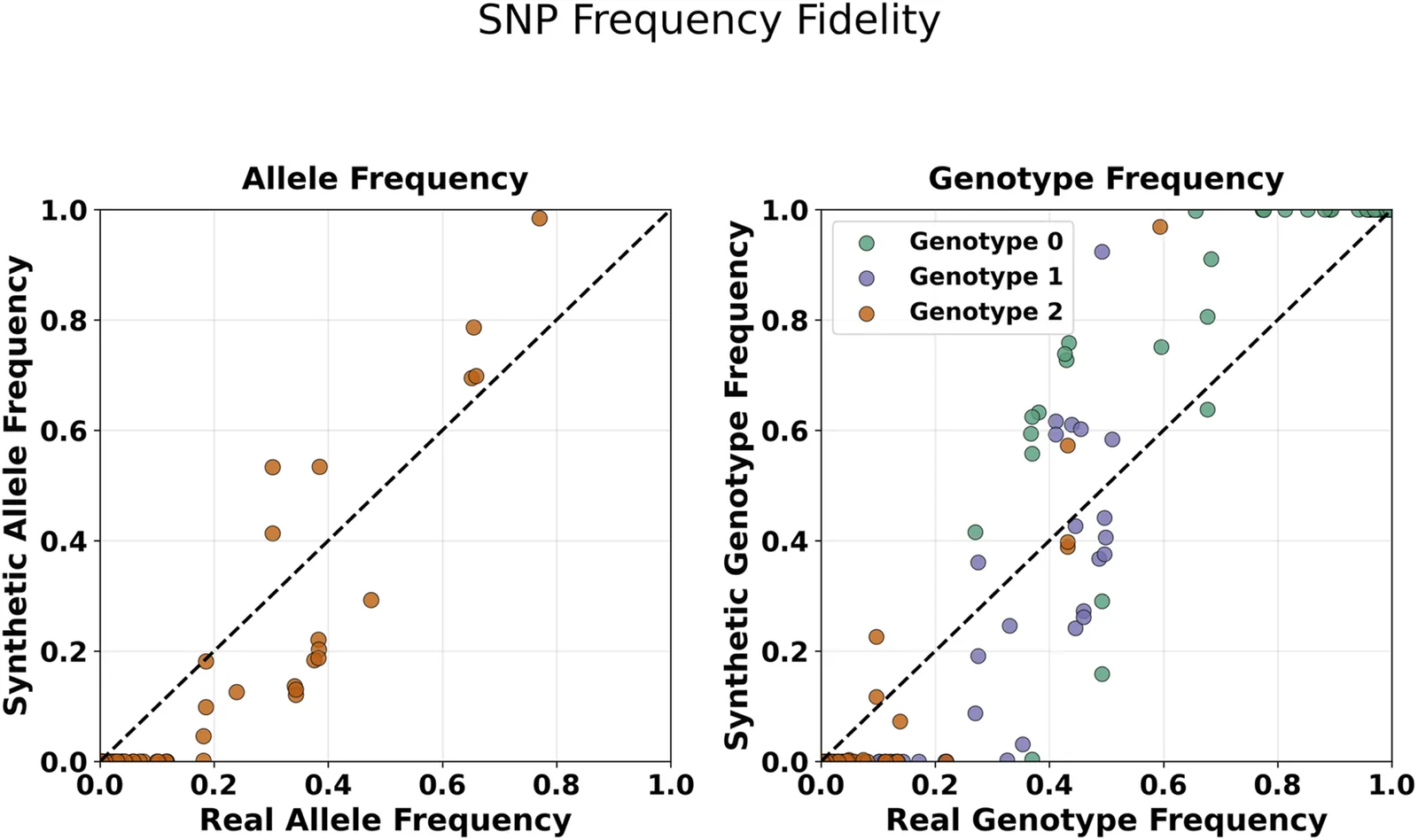
Real versus synthetic SNP allele and genotype frequencies. The left panel compares allele frequencies between the real and synthetic data, and the right panel compares genotype frequencies by genotype state. The dashed line indicates perfect alignment between real and synthetic frequencies (y = x). Points on the x-axis represent variants that became monomorphic in the synthetic data, meaning that variation at those SNPs was lost during generation.

As can be seen from Figure 8, points overlap along the horizontal axis when the allele frequencies are close to 0, indicating that SNPs with nonzero frequencies in the real data correspond to frequencies near 0 in the synthetic data. For these SNPs, the model assigned the same genotype to nearly all synthetic samples. Variant capture decreased as MAF declined (Table 5). Most variants that were not retained were rare or ultra-rare (MAF <1%) and were represented by only a few minor alleles in the development cohort. This limited representation likely made their genotype distributions more difficult for the model to learn accurately. Overall, TabDDPM preserved common genetic variation more consistently than rare variation in this cohort.

To assess possible exact copying of real genotype profiles, we compared each synthetic sample’s complete 168-SNP genotype vector with the genotype vectors of all 433 real development cohort participants and found that none of the synthetic samples exactly matched a real participant’s vector. This finding indicates that no exact duplication of complete genotype vectors was detected in the generated dataset.

### 3.2 Model performance comparison

We evaluated whether adding synthetic samples to the real training data improved classification performance by comparing eight model configurations across augmentation ratios of 0×, 3×, 5×, 7×, and 10× (Table 6), where the ratio denotes the number of synthetic samples added per real sample and 0× corresponds to real data only. Mean AUC generally increased with augmentation across the nonlinear models. The categorical late-fusion model achieved the highest mean cross-validated AUC at 7× augmentation (0.781), compared with 0.738 using real data alone, corresponding to an increase of 0.043. After averaging the participant-level out-of-fold predictions across the 10 repeats, the 7× versus 0× comparison yielded a raw paired DeLong p-value of 0.0012; after Holm adjustment across the 32 prespecified augmented-versus-real-only comparisons, the adjusted p-value was 0.03. Because these predictions were averaged across repeated model fits, this DeLong result was interpreted as an internal cross-validation comparison. The paired bootstrap analysis on the independent held-out cohort provided the primary uncertainty assessment for the selected model.

**TABLE 6:** Cross-validated model performance with and without synthetic data augmentation. Values are reported as mean AUC (standard deviation) across 50 outer evaluation runs from stratified 5-fold cross-validation repeated 10 times within the 433-participant development cohort. Results are shown for eight model configurations across augmentation ratios of 0×, 3×, 5×, 7×, and 10×, where the ratio denotes the number of synthetic samples added per real sample and 0× corresponds to real data only. For each model, the 10 out-of-fold predictions for each development participant were averaged across repeats. Paired DeLong tests compared the resulting participant-level prediction vectors for each augmented condition with the corresponding 0× condition. P-values were adjusted using the Holm procedure across the resulting 32 comparisons. The categorical late-fusion model achieved the highest mean AUC (0.781) at 7× augmentation.

Model	Encoding	Mean AUC (SD) by augmentation ratio				
		0×	3×	5×	7×	10×
Ridge logistic regression (L2 penalty; primary linear baseline)	Numeric	0.668 (0.045)	0.665 (0.047)	**0.670 (0.048)**	0.667 (0.049)	0.663 (0.051)
Unregularized logistic regression (no penalty; reference model)	Numeric	0.661 (0.058)	0.659 (0.060)	**0.666 (0.061)**	0.663 (0.062)	0.660 (0.063)
XGBoost	Numeric	0.714 (0.048)	0.722 (0.052)	0.731 (0.055)	0.735 (0.057)	**0.736 (0.058)**
CatBoost	Categorical	0.710 (0.051)	0.724 (0.053)	**0.741 (0.056)**	0.738 (0.057)	0.733 (0.060)
Early fusion	Numeric	0.660 (0.052)	0.667 (0.051)	**0.683 (0.054)**	0.680 (0.053)	0.675 (0.056)
Early fusion	Categorical	0.690 (0.049)	0.706 (0.051)	0.721 (0.052)	**0.725 (0.050)**	0.722 (0.054)
Late fusion	Numeric	0.718 (0.055)	0.733 (0.054)	0.743 (0.055)	**0.751 (0.056)**	0.749 (0.058)
Late fusion	Categorical	0.738 (0.053)	0.754 (0.051)	0.773 (0.055)	**0.781 (0.052)**	0.779 (0.054)

Selected comparison, categorical late fusion 7× vs. 0×: raw paired DeLong p = 0.0012; Holm-adjusted p = 0.03. Bold values indicate the highest mean AUC for each model across the augmentation conditions.

The unregularized logistic-regression model showed mean AUCs similar to those of Ridge logistic regression at the 0×, 5×, and 10× conditions (0.661 vs. 0.668, 0.666 vs. 0.670, and 0.660 vs. 0.663, respectively). These small differences suggested that the linear baseline showed little sensitivity to the choice of regularization.

The augmentation ratio yielding the highest mean AUC varied by model: 5×, 7×, or 10×. Performance did not increase monotonically as more synthetic data were added. Instead, gains flattened and, in some cases, declined at higher ratios. Because differences between adjacent ratios were small relative to cross-validation variability, the main finding is that moderate augmentation improved performance, rather than that any single ratio was universally optimal. The categorical late-fusion model showed comparable performance across the higher augmentation ratios, with the highest mean AUC observed at 7×. We therefore selected 7× as a representative high-performing setting for subsequent held-out evaluation rather than as a universally optimal augmentation ratio.

To assess whether the primary findings were consistent across alternative feature sets and resampling strategies, we conducted additional comparison analyses using the same 50 outer cross-validation runs (Table 7). The demographics-only L2-regularized logistic regression achieved a mean AUC of 0.701, while the SNP-only L2-regularized logistic regression achieved a mean AUC of 0.716, suggesting that both demographic and genetic features contained predictive information. Because the primary model used categorical late fusion, these results do not quantify the independent contribution of either feature group to the final model. As a non-generative class-rebalancing control, RandomOverSampler achieved a mean AUC of 0.744 using the same categorical late-fusion architecture, lower than the 0.781 achieved with TabDDPM augmentation. Because RandomOverSampler was not sample-size matched and no formal statistical test was performed, this comparison was descriptive.

**TABLE 7:** Additional feature-set and class-rebalancing analyses. Values are mean AUC (SD) across the 50 repeated outer cross-validation runs. The first two rows evaluate L2-regularized logistic regression using demographic-only and SNP-only inputs. The remaining rows evaluate the categorical late-fusion model under real-data-only, RandomOverSampler, and 7× TabDDPM training conditions.

Model	Mean AUC (SD)
Demographics-only + L2 logistic regression	0.701 (0.046)
SNPs only + L2 logistic regression	0.716 (0.051)
Categorical late fusion + real data only (0×)	0.738 (0.053)
Categorical late fusion + RandomOverSampler	0.744 (0.049)
Categorical late fusion +7× TabDDPM	0.781 (0.052)

Under 7× TabDDPM augmentation, the categorical late-fusion model showed similar performance across embedding dimensions of 4, 8, and 16, with mean AUCs of approximately 0.781, 0.781, and 0.782, respectively (Table 8). These results suggest that performance was not sensitive to embedding size within the tested range and the four-dimensional embedding provided a similarly performing compact representation.

**TABLE 8:** Sensitivity of the categorical late fusion model to embedding dimension under 7× TabDDPM augmentation. Values are mean AUC (SD) across the 50 outer cross-validation runs.

Embedding dimension	Mean AUC (SD)
4	0.781 (0.052)
8	0.781 (0.052)
16	0.782 (0.053)

Figure 9 illustrates a representative cross-validation run in which the 7× model’s AUC was closest to the mean across the 50 runs. The augmented model had a larger area under the ROC curve than the real-only model. The curves overlapped at low false-positive rates but separated more clearly at moderate to high false-positive rates, consistent with the higher mean AUC observed with augmentation.

**FIGURE 9:**
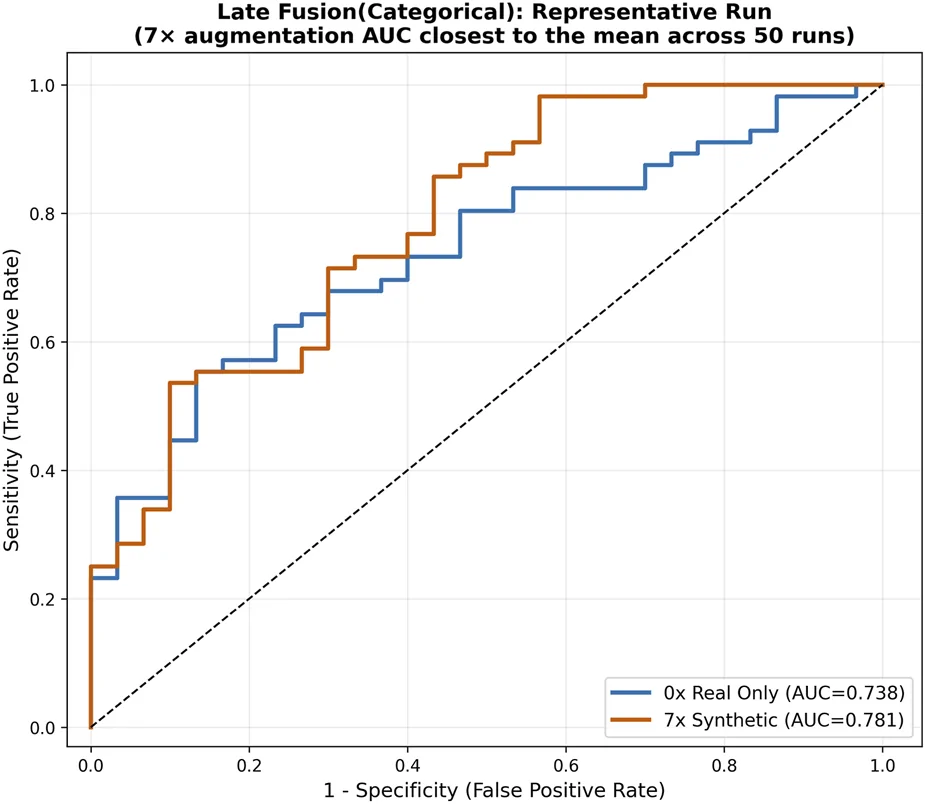
ROC curves for the categorical late fusion model trained on real data alone (0×, blue) and with 7× synthetic augmentation (orange). Curves are shown for the representative cross-validation run, selected as the run in which the 7× model’s AUC was closest to its mean AUC across the 50 runs. The displayed curves therefore do not represent mean ROC curves. The curves plot sensitivity against the false-positive rate (1 − specificity) across classification thresholds. The dashed diagonal denotes chance-level performance. The reported mean AUCs across all 50 runs were 0.738 for real-data-only training and 0.781 with 7× augmentation.

### 3.3 Held-out evaluation

To evaluate whether the improvement observed during cross-validation extended to unseen participants, we compared the performance and confusion matrices of the categorical late-fusion model trained with real data alone and with 7× synthetic augmentation on the 109-participant held-out cohort (Table 9; Figure 10).

**TABLE 9:** Held-out performance of the categorical late fusion model with and without synthetic augmentation. Values are reported as the mean (standard deviation) across 20 retraining seeds on the 109-participant held-out cohort (37 cognitively normal, 72 cognitively impaired). Standard deviations represent training variability across seeds. The final row reports paired augmented-minus-real differences with 95% BCa confidence intervals based on participant-level bootstrap resampling.

Model	Held-out cohort mean (SD)		
	AUC	Sensitivity	Specificity
0× (real data only)	0.721 (0.018)	0.68 (0.034)	0.58 (0.039)
7× augmentation	0.769 (0.012)	0.77 (0.027)	0.66 (0.031)
Paired difference (95% BCa CI)	+0.048 [+0.030, +0.080]	+0.09 [+0.06, +0.12]	+0.08 [+0.02, +0.11]

**FIGURE 10:**
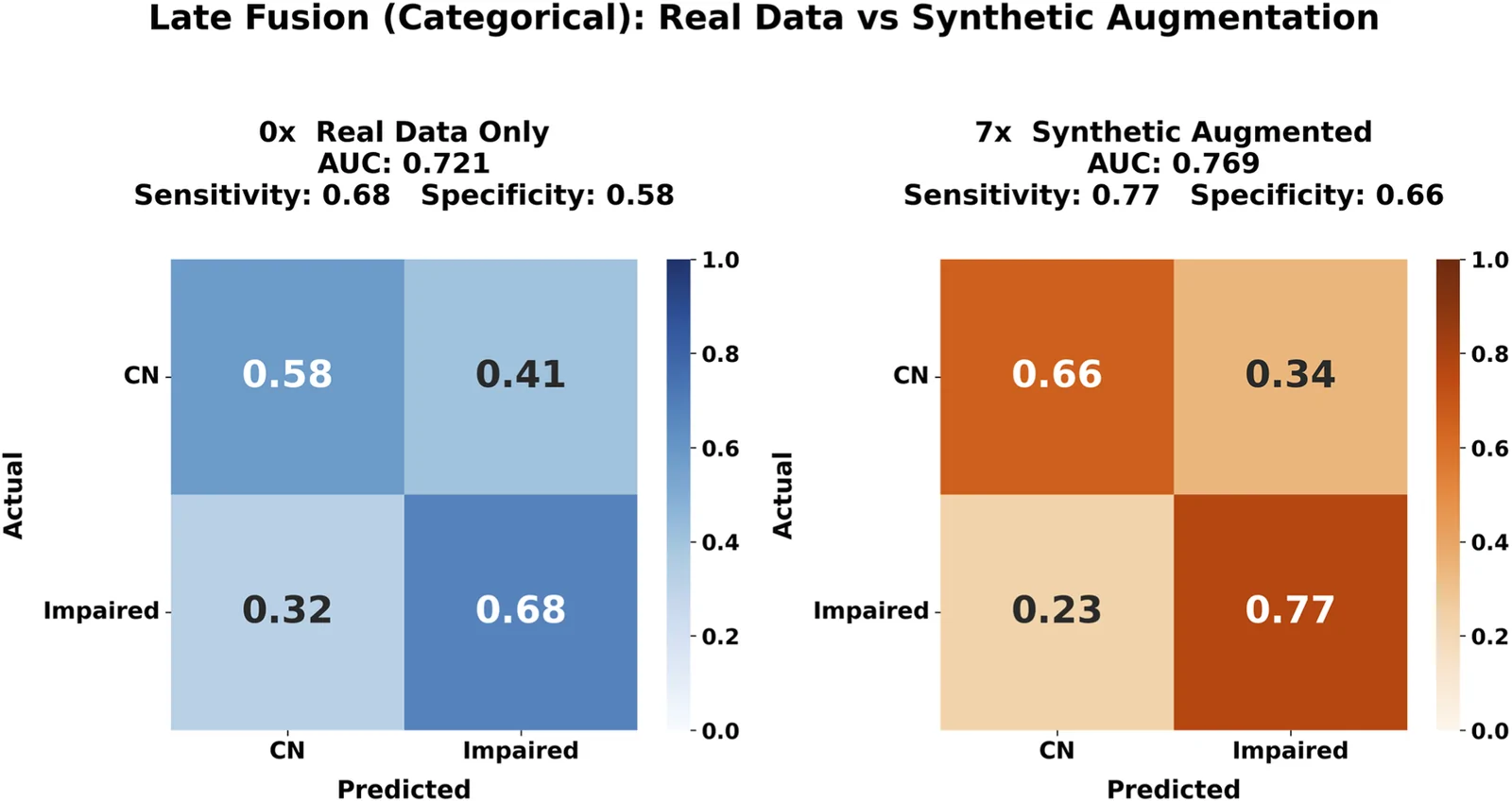
Confusion matrices for the categorical late-fusion model on the 109-participant held-out cohort (37 cognitively normal and 72 cognitively impaired participants), trained with real data alone (0×; AUC = 0.721, left) or with 7× synthetic augmentation (AUC = 0.769, right). Cell values are row-normalized proportions averaged across 20 retraining seeds and therefore do not represent a single integer confusion matrix. Diagonal entries represent mean specificity and sensitivity. Condition-specific thresholds were selected using Youden’s J statistic from development cohort out-of-fold predicted probabilities and fixed before held-out evaluation.

Trained on real data alone, the categorical late-fusion model achieved a mean held-out AUC of 0.721, sensitivity of 0.68, and specificity of 0.58 across 20 retraining seeds. With 7× synthetic augmentation, mean held-out AUC increased to 0.769, sensitivity to 0.77, and specificity to 0.66 (Figure 10). The standard deviations across the 20 runs primarily reflect variability across model retraining seeds rather than participant-level uncertainty.

The development-derived Youden thresholds were 0.51 for the real-only condition and 0.47 for the 7× augmented condition. Because classification thresholds were selected separately for the two conditions, the corresponding sensitivity and specificity values reflect performance at different operating points.

Participant-level paired bootstrap analysis based on 5,000 stratified resamples estimated an AUC difference of +0.048 (95% BCa [+0.030, +0.080]), a sensitivity difference of +0.09 (95% BCa [+0.06, +0.12]) and a specificity difference of +0.08 (95% BCa [+0.02, +0.11]). All three BCa confidence intervals excluded zero, supporting improvements in discrimination, sensitivity, and specificity with augmentation.

The sensitivity increase (+0.09) indicates that fewer cognitively impaired participants were classified as cognitively normal. Specificity also increased (+0.08), although its confidence interval was wider and its lower bound was closer to zero, indicating greater uncertainty than for the sensitivity improvement.

The 109 held-out participants were not used for TabDDPM training or tuning, predictive model selection, or predictive model training. The improvement observed in this cohort therefore provides a separate held-out assessment of the selected augmentation strategy. The held-out AUC increased by 0.048, which was directionally consistent with the 0.043 increase observed during cross-validation.

## 4 Discussion and conclusion

Deep learning methods have shown strong performance in many genetic and genomic prediction tasks. However, their use in small clinical genomic cohorts remains challenging since these cohorts often contain only a few hundred participants with genotype, demographic, and diagnostic data. This sample size is usually too small for neural networks to learn stable nonlinear patterns without overfitting. In this paper, we propose an approach that uses TabDDPM-generated synthetic data to augment the training of deep learning models. On real ADNI data, we found that synthetic data augmentation can improve classification performance in small clinical genomic cohorts, broadening the potential application of deep learning to clinical genomic classification when patient data is limited.

The main finding is that synthetic augmentation improved performance across nonlinear models. Across the four neural network configurations, the maximum increases in mean cross-validated AUC ranged from 0.023 to 0.043. The strongest result was achieved by the categorical late fusion network, which improved from 0.738 using real data alone to 0.781 at 7× augmentation. XGBoost and CatBoost also showed improvements with augmentation. This pattern suggests that synthetic data may have helped the nonlinear models learn patterns that were difficult to extract from the real cohort alone.

Synthetic augmentation appears to make the existing signal easier for the model to learn. With 433 real participants, the available predictive signal is limited, and nonlinear models can overfit patient-specific variation rather than learn stable genotypes and demographic patterns. The synthetic records are derived from the real participants and should not be interpreted as new independent observations. They may smooth the training distribution, provide additional plausible feature combinations, or act as a form of regularization. These effects may reduce overfitting to participant-specific variation and help nonlinear models learn a more stable decision boundary.

At 7× augmentation, ridge logistic regression showed essentially no change in mean AUC (0.668–0.667), whereas the categorical late-fusion model increased from 0.738 to 0.781. This contrast is consistent with the possibility that synthetic augmentation was more beneficial for models capable of representing complex relationships among demographic and genotype features. However, because the models also differed in architecture and genotype representation, these results do not establish that nonlinear genetic interactions themselves caused the observed performance difference.

The benefit of augmentation depends on the synthetic-to-real ratio. Performance generally improved after adding synthetic samples and then leveled off between 5× and 10× augmentation. The gains were not monotonic, and higher augmentation ratios reduced performance for some models, suggesting that excessive synthetic data can introduce noise. We selected the 7× ratio because it gave the best performance for the categorical late fusion model and fell within the broader range where augmentation was consistently useful. It should be viewed as an effective setting for this cohort, not as a universal optimum. In fact, it has been shown in Ma and Zhang (2026) that synthetic augmentation is not always beneficial and that its optimal amount depends on generator quality and the learning setting, with excessive augmentation potentially degrading performance. Our empirical results showed a similar pattern.

The neural network architecture also affected performance. Late fusion outperformed early fusion for both numerical and categorical genotype encodings. Early fusion combined the three demographic and 168 genotype features at the input layer, whereas late fusion processed them in separate branches before combining their representations. Separate processing may have helped the model use demographic and genotype features effectively in this cohort. These results suggest that separate branches may be useful when combining a small number of demographic features with a larger genomic feature set, although the observed advantage was specific to this cohort and feature set.

Categorical genotype embeddings also improved performance compared with numerical encoding. An embedding treats each genotype state as a distinct category. This could allow the model to learn non-additive genotype effects, which are biologically reported in the *APOE* region. This benefit was obtained with a four-dimensional embedding. Performance was similar across embedding sizes of 4, 8, and 16. Given the relatively small 168-SNP feature set, these results suggest that a compact embedding was adequate to capture the genotype-state structure within the tested range.

The fidelity analysis showed that TabDDPM reproduced key cohort-level demographic features, even in a limited-data setting. Age and education distributions were well matched between the real and synthetic data, and gender composition within diagnostic groups was preserved. Because the diagnostic labels were specified during class-conditional generation, the close match in diagnostic class proportions reflects the sampling design rather than learned fidelity. It confirms that the conditional generation was applied correctly. These results indicate that the synthetic data retained demographic patterns that may be relevant to classification.

For SNP features, TabDDPM reproduced common variants more reliably than rare variants. Among variants with a minor allele frequency of at least 5%, 84% remained polymorphic in the synthetic data, indicating that common genetic variation was generally preserved. Rare variants, however, were reproduced less consistently. Among SNPs with a minor allele frequency below 1%, none retained variation in the synthetic data. These variants were represented by only a small number of minor alleles in the 433-participant development cohort, providing limited information from which the generative model could learn their distributions. Overall, these findings indicate that TabDDPM preserved common genetic variation more effectively than rare variation in this cohort.

Our findings demonstrate both the potential and the limitations of diffusion-based augmentation in a cohort of this size. One possible explanation for the augmentation benefit is that TabDDPM preserved common genotype variation more consistently than rare variation. Rare-variant information was not recovered reliably when it was scarcely represented in the source data. The more consistent preservation of common variants may have contributed to the improvement in downstream performance observed in this cohort. Further gains, however, will likely require broader genomic features rather than generating more synthetic samples. Fidelity analyses were limited to comparisons of marginal SNP frequencies and exact genotype-vector duplication checks. These findings should therefore be interpreted as descriptive rather than as a comprehensive assessment of synthetic-data fidelity. More formal distributional analyses would strengthen future work.

Sensitivity increased from 0.68 to 0.77 and specificity from 0.58 to 0.66 with synthetic augmentation. Because classification thresholds were selected separately for the real-only and augmented conditions, these sensitivity and specificity values reflect performance at different operating points and should not be interpreted as a direct fixed-threshold comparison. Held-out AUC, which is threshold-independent, increased from 0.721 to 0.769, with a paired participant-level difference of +0.048 (95% BCa [+0.030, +0.080]). This provides the most direct evidence of improved discrimination between cognitively normal and cognitively impaired participants.

Because the cross-validation DeLong analysis used participant-level out-of-fold predictions averaged across 10 repeats, its p-values may not fully capture variability from repeated model fitting and training partitions. The paired bootstrap analysis on the held-out cohort provided a separate assessment of uncertainty for the selected model.

This study used one ADNI cohort. The synthetic data were generated from the same source cohort, so augmentation cannot correct biases or supply biological variation absent from the original data. The feature set was also limited to *APOE*-region SNPs and three demographic variables. Validated *APOE* ε-genotype definitions and externally derived polygenic risk score (PRS) weights were not available for the prespecified 168-SNP *APOE*-region panel. Therefore, the study does not establish superiority over ε-genotype or PRS reference models. This design allowed us to test the augmentation strategy, but it does not capture the full genetic and biological complexity of Alzheimer’s disease. A further limitation was the small number of AD participants in the development cohort (n = 25), which motivated combining MCI and AD into a single cognitively impaired class. Although this binary formulation improved the stability of model development and evaluation, it may obscure differences in impairment severity between MCI and AD. The modeling framework itself is not restricted to binary classification and could be extended to distinguish CN, MCI, and AD separately in larger cohorts with sufficient representation of each diagnostic group.

Because RandomOverSampler was used as a class-rebalancing rather than a sample-size-matched control, and no formal statistical test was performed, this comparison should be interpreted as descriptive and does not fully isolate the contribution of generative augmentation from the effects of differing numbers of added training samples.

Our results demonstrate that generative augmentation is a promising strategy for augmenting cohorts with limited sample size to support neural network models. Because this approach uses standard tabular genomic and demographic features rather than disease-specific inputs, it can be adapted to other classification tasks with minimal modification. Therefore, the contribution of this study is not limited to cognitive impairment classification in Alzheimer’s disease. It also provides a methodological framework for developing classification models in many clinical domains where individual-level data are limited.

## Data Availability

Data used in this study were obtained from the Alzheimer’s Disease Neuroimaging Initiative (ADNI) database (adni.loni.usc.edu). The code used for this research is available on GitHub at github.com/aw204/AD-Classification-Research.
